# CRISPR/Cas9-Based Genome Editing and Its Application in *Aspergillus* Species

**DOI:** 10.3390/jof8050467

**Published:** 2022-04-30

**Authors:** Feng-Jie Jin, Bao-Teng Wang, Zhen-Dong Wang, Long Jin, Pei Han

**Affiliations:** 1 Co-Innovation Center for Sustainable Forestry in Southern China, College of Biology and the Environment, Nanjing Forestry University, 159 Longpan Road, Nanjing 210037, China; jinfj@njfu.edu.cn (F.-J.J.); wbt@njfu.edu.cn (B.-T.W.); zdwang98@163.com (Z.-D.W.); isacckim@kaist.ac.kr (L.J.); 2 Technology and Engineering Center for Space Utilization, Key Laboratory of Space Utilization, Chinese Academy of Sciences, 9 Deng Zhuang South Rd, Beijing 100094, China

**Keywords:** *Aspergillus* species, genome editing technology, CRISPR/Cas9, cell factory, natural product production

## Abstract

*Aspergillus*, a genus of filamentous fungi, is extensively distributed in nature and plays crucial roles in the decomposition of organic materials as an important environmental microorganism as well as in the traditional fermentation and food processing industries. Furthermore, due to their strong potential to secrete a large variety of hydrolytic enzymes and other natural products by manipulating gene expression and/or introducing new biosynthetic pathways, several *Aspergillus* species have been widely exploited as microbial cell factories. In recent years, with the development of next-generation genome sequencing technology and genetic engineering methods, the production and utilization of various homo-/heterologous-proteins and natural products in *Aspergillus* species have been well studied. As a newly developed genome editing technology, the clustered regularly interspaced short palindromic repeats/CRISPR-associated protein 9 (CRISPR/Cas9) system has been used to edit and modify genes in *Aspergilli*. So far, the CRISPR/Cas9-based approach has been widely employed to improve the efficiency of gene modification in the strain type *Aspergillus nidulans* and other industrially important and pathogenic *Aspergillus* species, including *Aspergillus oryzae*, *Aspergillus niger*, and *Aspergillus fumigatus*. This review highlights the current development of CRISPR/Cas9-based genome editing technology and its application in basic research and the production of recombination proteins and natural products in the *Aspergillus* species.

## 1. Introduction

Filamentous fungi play a critical role in human health and disease, as well as in industrial and agricultural production. *Aspergillus* sp. is one of the most widely disseminated genera of fungi in nature, releasing a large number of conidia and dispersing them across the environment, including in grain, soil, and organisms. The genus *Aspergillus* is comprised of over 300 species based on morphological, physiological, and phylogenetic characteristics that have a considerable impact on food production, biotechnology, environments, and human health. [1]. This genus encompasses a large number of species that occupy an essential ecological niche in natural habitats as decomposers and pathogens. *Aspergillus* species has long been recognized as an important environmental microorganism in the breakdown of organic materials in terrestrial ecosystems [2]. Meanwhile, some species of the genus *Aspergillus* play key roles in the traditional fermentation and food processing industries due to their remarkable ability to produce a huge quantity of hydrolytic enzymes and other natural products and can thus be employed as microbial cell factories. For example, some *Aspergillus* strains, such as *Aspergillus niger* and *Aspergillus oryzae*, have been well utilized to produce a variety of beneficial substances, including citric acid, sake brewing, soy sauce, and so on [3,4,5]. Because of their long-term use in the food industry, both *A. oryzae* and *A. niger* are listed as Generally Recognized as Safe (GRAS) organisms; the non-pathogeny of *A. oryzae* is also supported by the Food and Agriculture Organization/World Health Organization (FAO/WHO) [6].

*A. oryzae* has a high capacity for secreting huge numbers of hydrolytic enzymes and, therefore, it has been used as a cell factory in the enzyme industry to produce a variety of native and heterologous enzyme preparations [7,8,9]. Furthermore, *A. niger* is also a vital industrial production strain, with organic acids and industrial enzyme preparations being commonly produced [10,11,12]. Within the genus, *Aspergillus nidulans* has received widespread recognition as a model eukaryote in fungal fundamental research because its morphology, physiology, and growth conditions have been well characterized; in the meanwhile, it is a potential resource and is frequently employed in the production of industrial enzymes [13,14]. These *Aspergillus* strains have the advantages of easy culture, fast growth, and strong synthetic capacity; therefore, they are also well used in the production of other valuable natural products [15,16,17,18,19,20]. In *A. nidulans*, for example, more than 30 biosynthetic gene clusters have been identified to be associated with specific natural products, although half of them remain uncharacterized [21]. Aside from these, several *Aspergillus* spp. are also involved in human health and disease. For example, *Aspergillus flavus* produces aflatoxin, a carcinogen [22,23], and *Aspergillus fumigatus* causes aspergillosis [24,25]. In studies, these strains have been reported to harm the gut and respiratory organs of cattle, poultry, and even humans. Globally, *Aspergillus* is estimated to be responsible for over 200,000 invasive aspergilloses (IA) cases annually, the majority of which are caused by *A. fumigatus* [26]. As the most prevalent airborne fungal pathogenic species found in nature, *A. fumigatus* is becoming an increasingly lethal threat to immunocompromised individuals. Despite the fact that information on the *A. fumigatus* genome sequencing is available through online genomics resources, a large number of genes that may be involved in pathogenicity remain poorly understood. The increasing number of entire genomes sequenced from various fungal species, including *Aspergillus* spp., has raised the bar for genetic modification in the study of filamentous fungi [27,28,29]. Recent advances in genetic manipulation techniques, such as the development of various selective markers, improved transformation efficiency, and improved gene deletion efficiency, among others [30,31,32], have greatly facilitated these basic studies and breeding for industrial production [33,34,35]. However, these genetic manipulation techniques still require a significant amount of labor and time to prepare the host/vector systems for each industrial strain for further industrial production. Recently, the clustered regularly interspaced short palindromic repeat (CRISPR)/CRISPR-related nuclease 9 (Cas9) system, a versatile genome-editing technique that may give more precise gene modification, has been extensively developed and employed in a wide range of fields of filamentous fungi [36,37,38]. With its advantages of simple manipulation, targeted specificity, high-efficiency single/multiple-gene editing, and a wide range of versatility, the CRISPR/Cas9-based genome editing approach has been well applied to various *Aspergillus* species.

In this review, the overall technological advancements of CRISPR/Cas9-based genome editing strategies and their applications in basic research and the production of recombinant proteins and natural products in *Aspergillus* spp. are outlined and explored.

## 2. CRISPR/Cas9-Based Genome Editing in *Aspergillus* Species

From prokaryotes to eukaryotes, the CRISPR/Cas system has been proven to effectively modify genes in virtually all species [37,39,40]. The CRISPR/Cas system could be classified into two categories and six major types [41,42]. Currently, as a simpler CRISPR system, the type II CRISPR/Cas9 system has been widely used in different species. The type II system is comprised of nuclease (Cas9), mature CRISPR RNA (crRNA), trans-activating crRNA (tracrRNA), and RNaseIII. In addition, the crRNA can combine with the tracrRNA to generate a single-guide RNA (sgRNA) complex [43], which may effectively induce the Cas9 nuclease to cleave the target sequences.

DNA double-strand breaks (DSBs) in eukaryotes can be repaired by two DNA self-repair mechanisms: the non-homologous end-joining (NHEJ) and homologous directed repair (HDR) pathways. When DSBs occur, the genomic DNA initiates its repair process, and as the dominant repair pathway, the NHEJ can lead to genomic alteration by causing random loss, insertion, and replacement of bases at DSB locations. The HDR pathway, on the other hand, allows for precise editing of target genes with the use of exogenous donor DNA [36,44].

CRISPR/Cas9-based genome editing technology is currently being employed extensively in filamentous fungi, including numerous vital genera, such as *Neurospora*, *Trichoderma*, *Penicillium*, and *Aspergillus* [45,46,47,48]. This genome editing strategy and its application have been well established, particularly in *Aspergilli*.

### 2.1. Cas9 Expression

The CRISPR/Cas9 system was first discovered as an immunological defense system in bacteria and archaea. With a total length of approximately 1400 amino acids, the Cas9 protein originating from the bacteria *Streptococcus pyogenes* is a critical component of this genome editing machinery that functions as a nuclease [49]. When CRISPR/Cas9-based genome editing is applied to filamentous fungi, the Cas9-encoding gene usually needs to be codon-optimized and fused with a nuclear localization signal for its correct expression and localization [48,50]. An identified SV40 nuclear localization sequence (NLS; PKKKRKV) has been effectively exploited to guide the nuclear localization of Cas9 in many filamentous fungi, such as the genus *Aspergillus* [48,51].

Furthermore, another major factor determining the expression efficiency of the *cas9* heterologous gene is the promoter employed for Cas9 protein production. Therefore, choosing the right promoters is crucial for the CRISPR/Cas9 system to work properly. Several commonly used promoters have been successfully applied to drive the expression of Cas9 protein in *Aspergillus* species, such as the promoters of *tef1* (translation elongation factor 1-alpha gene), *gpdA* (glyceraldehyde-3-phosphate dehydrogenase gene), *amyB* (α-amylase gene), and *glaA* (glucoamylase gene) [48,50,52,53,54] (Table 1). In addition to these typical promoters, P*pkiA*, P*coxA*, and other promoters have also been effectively employed to express Cas9 in *Aspergillus species* [52,55]. Cas mutants and their fusion with other functional proteins may be able to extend genome editing capabilities even further. Previous research has demonstrated that, in mammalian cells, a unique approach called “base editor” has been developed that avoids DNA damage during genome editing and does not require the provision of an HDR donor template [56]. It has been confirmed that a catalytic mutant of SpCas9 (D10A nickase) may facilitate gene editing via HDR without NHEJ-induced insertion-deletion formation [49]. The Cas9 mutant (D10A nickase) is used in combination with a rat cytidine deaminase and uracil glycosylase inhibitor to convert cytidine (C) to uridine (U) at the target sites. This base editing technique has been successfully applied in *Aspergillus*, where it can directly edit single nucleotides and cause high-frequency C→T replacement [57].

The specific cleavage site of Cas protein in the genome depends on both the guide RNA (gRNA) and the protospacer adjacent motif (PAM). At present, the PAM sequence of SpCas9 from *S. pyogenes* commonly used in *Aspergillus* is 5′-NGG-3′ [49]. Unlike bacteria, most filamentous fungi lack native extra chromosome replicating DNA elements, such as plasmids. However, studies have shown that AMA1 (autonomously maintained in *Aspergillus*) derived from *A. nidulans* can be used to construct autonomous replicating plasmids, which are often employed to express Cas9 and sgRNA. Multiple copies of AMA1-carrying plasmids within a cell may lead to increased expression of Cas9 and sgRNA, thus boosting the mutation efficiency of CRISPR/Cas9-mediated genome editing [52,58]. Furthermore, when the plasmid also carried a *pyrG* selective marker, the AMA1-based plasmid may be easily removed in the presence of 5-fluoroorotic acid and uridine, allowing the *pyrG* and Cas9 components to be recycled [59]

### 2.2. Guide RNA Expression

The CRISPR/Cas9 system is an RNA-guided nuclease system that can efficiently execute sequence-specific DNA cleavage. The gRNA in the natural CRISPR/Cas system of bacteria or archaea consists of two regions: a CRISPR RNA (crRNA) harboring a target recognition sequence of 20-nucleotide at the 5′-end and a trans-activating crRNA (tracrRNA) for Cas9 engagement. In the presence of endogenous RNase III, the tracrRNA guides the precursor crRNA to be processed into mature crRNA [60]. To generate genetic mutations through CRISPR, the gRNA can provide sequence specificity to the target DNA, which forms an RNA/DNA hybridization and recruits the Cas9 nuclease to cause DSB at the target genomic locus.

gRNA is often driven by endogenous RNA polymerase III *U6* promoters in most organisms, and these promoters exhibit base-preference and persistence in the transcriptional process [61]. The *U6* is known to be the most highly conserved small nuclear RNA (snRNA), and its promoter has been exploited for gRNA transcription in filamentous fungus, including *Aspergillus* [48,51]. Another RNA polymerase III *U3* promoter was also successfully used for gRNA transcription in *A. nidulans* [50,62]. In addition, the transfer ribonucleic acid gene (*tRNA*) promoters have also been well used for gRNA expression [55]; however, unlike the *U6* promoter, the genome-editing efficiency driven by different *tRNA* promoters varies greatly between strains. In addition to these, a high-efficiency promoter of the 5S rRNA gene, which is highly conserved and efficiently expressed in eukaryotes, was discovered and exploited as a gRNA promoter for CRISPR/Cas9 genome editing in *A. niger* [54]. This study used the 5S rRNA gene combined with its 338-bp upstream sequence as a promoter to fuse with gRNA sequence for gRNA expression. The results demonstrated that the 5S rRNA promoter has a greater gene disruption efficiency than those of *U6* and other promoters, and the CRISPR/Cas9 system based on the endogenous 5S rRNA promoter showed a gene disruption efficiency of nearly 96%. Recently, using the ribozyme self-processing capacity, the RNA polymerase II promoter was exploited for sgRNA expression through a ribozyme-gRNA-ribozyme gene system to synthesize mature sgRNA. According to this, a powerful constitutive *gpdA* promoter (P*gpdA*) from *A. nidulans* was used to construct a gRNA expression cassette, which was successfully applied to *Aspergillus* species [50,63].

Thus far, two kinds of CRISPR/Cas9 systems have been exploited for use in *Aspergillus* genetic engineering. The first is a plasmid vector expressing system, which contains the elements for expressing the Cas9 and gRNA in vivo, as previously stated; the second is a plasmid-free CRISPR/CAS9 approach, which has also recently been developed and is well adapted for genetic alteration [11,64,65]. In the plasmid-free CRISPR/Cas9 system, the Cas9 protein and sgRNA can be assembled in vitro to generate a stable Cas9/sgRNA ribonucleoprotein (RNP) complex, and then transferred into fungal protoplasts for genome editing via PEG or other transformation methods. The RNP-based method accurately controls the concentrations of the purified Cas9 protein and synthetic gRNA for in vitro assembly, thereby reducing the risk of off-target events. However, although RNP complexes may be utilized directly for genome editing, the approach lacks a selective marker for fungal transformation. Therefore, in some cases, an additional vector harboring a selective marker gene needs to be provided [11]. Recently, the RNP-based CRISPR/Cas9 system was successfully applied to *Aspergillus*, resulting in a marked increase in succinic acid production in the *A. niger*-engineered strain [11].

### 2.3. Donor DNA

Cas9-induced DSBs can either be directly subjected to NHEJ-mediated repair that generates insertion/deletion mutagenesis or can be repaired by HDR by providing a DNA repair template (donor DNA) to the target site for homologous recombination (HR). The NHEJ repair pathway is completely distinct from the HDR repair system in that it can introduce non-specific insertions or deletions at the cleavage site by directly connecting the ends of DNA DSBs, whereas the HDR pathway allows a precise gene editing that only occurs during DNA replication. During DNA damage repair, the provision of homologous DNA fragments might greatly improve gene targeting and repair efficiency via HR. In addition, the Ku70, Ku80, and LigD proteins are known to play essential roles in the NHEJ repair pathway, and the deletion of genes encoding these proteins leads to dramatically improved HR efficiency [30,66,67]. This has been well combined with CRISPR/Cas9-based genome editing technology, which can significantly raise the efficiency of gene targeting when a donor DNA fragment is provided [48,68]. In *Aspergillus*, co-transformation of fungal cells with the genome editing plasmid and circular/linear donor DNA fragments enabled marker-free multiplex gene deletion or integration. Selectable markers or drug resistance markers added into the donor DNA, on the other hand, can further improve the effectiveness of CRISPR/Cas9-mediated *Aspergillus* genome engineering [26,53]. In summary, the CRISPR/Cas9 system allows precise gene editing via the HDR pathway by providing donor DNA, such as introducing a specific point mutation or precisely replacing a target sequence with a desired one by inserting a designed sequence into target sites [50,53,58].

### 2.4. Off-Target Effects in CRISPR/Cas9-Based Genome Editing

CRISPR/Cas9-mediated genome editing technology has been successfully used in a variety of biological studies due to its high specificity, relatively simple manipulation, and high efficiency, but its off-target effects have also attracted widespread attention. In general, the off-target effect of the CRISPR/Cas9 system is mostly due to the recognition specificity of Cas9/sgRNA complex to target genes on the genome. Cas9 nucleases, for example, can recognize and cleave the mismatched base of an untargeted sequence, causing serious off-target effects. The RNA-guided Cas9 nucleases could be highly active, even with imperfectly matched RNA-DNA interfaces in human cells [69], and the detected off-target sites harbored up to five mismatches for each gRNA. Therefore, how to reduce the off-target effects is a major concern in genome editing. Screening and exploiting Cas mutants with high recognition specificity, rational design, selection of sgRNAs, regulation of Cas protein and sgRNA expression level, and other ways, are currently being used to limit off-target effects. Firstly, an *S. pyogenes* Cas9 mutant (SPCas9-HF1) with high recognition specificity was constructed to avoid genome-wide off-targets. This mutant is designed to significantly reduce the non-specific DNA contacts with mismatched sequences while retaining on-target activities, thus reducing the risk of off-target [70]. Second, sgRNA design tools or off-target risk prediction software can be used to assess the specificity of the target sequence in the genome. For instance, sgRNAcas9, a software package, is available (www.biootools.com, accessed on 1 April 2022) for predicting the potential off-target cleavage sites and designing sgRNA to improve CRISPR-Cas9 specificity for targeted genome editing [71]. Previous studies also showed that high concentrations of Cas9/gRNA complexes could trigger off-target effects. Therefore, thirdly, studies attempted to regulate the expression levels of sgRNA and Cas proteins. Down-regulating the transcription and translation levels of sgRNA and Cas proteins in cells has been found to significantly reduce the risk of off-targets [72]. Recently, a CRISPR/Cas9 system designed exclusively for transient expression was further developed [73]. When the Cas9 protein and sgRNA are assembled in vitro to form a stable RNP complex and subsequently transform into the fungal cells, the off-target effects can also be reduced due to their instantaneous existence. In addition, RNP transformation minimizes the likelihood of genetic material being integrated into non-target regions of the genome. These strategies provide effective schemes for decreasing the off-target effects of genome editing, thereby improving the specificity of the CRISPR/Cas9 system in *Aspergillus* species.

## 3. Development and Application of CRISPR/Cas9-Based Genome Editing Technology in Several *Aspergillus* Species

*Aspergillus* fungus serves a critical role in the production of secreted proteins and the decomposition of organic matter, making them popular in the food fermentation industries and for recombinant protein production. Recently, they have also been widely used as hosts for the production of industrially valuable secondary metabolites. Despite the fact that the *Aspergillus* spp. have been used to manufacture a range of critical enzymes and/or natural metabolites, wild-type strains are often unable to produce the desired products at the industrial level. Therefore, genetic engineering techniques are utilized to further boost the productivity of these industrial strains, whereas traditional genetic manipulation approaches are time-consuming and laborious.

More recently, the CRISPR/Cas9-based genome editing technique has been well applied in the basic research and manufacturing applications of natural products and recombinant proteins in the genus *Aspergillus* (Table 1) [74,75].

### 3.1. Aspergillus Nidulans

The *Aspergillus* species is considered as a suitable host for industrial enzyme production because of its high secretion capacity and safety. *A. nidulans*, as a type strain in the genus *Aspergillus*, plays a key role in basic fungal research; meanwhile, it has also been widely applied in the production of industrial enzymes and natural products [13,21]. The CRISPR/Cas9 system was first established for genetic engineering in *Aspergilli*, including *A. nidulans*, *A. niger*, *A. aculeatus*, and others, by Nodvig et al. [63]. In this study, mutations in the *yA* gene, which can change the color of spores, were utilized to investigate the efficiency of this genome editing. Following that, Cpf1, a new tool originating from *Lachnospiraceae bacterium*, was employed to replace the Cas9 nuclease in the fungal CRISPR technology [62]. The codon-optimized *Lb_cpf1* nuclease mediated CRISPR experiments have shown that Cpf1 can be used effectively for gene editing in *Aspergilli*. Recent studies have also shown that CRISPR-mediated transcriptional activation of fungal biosynthetic gene clusters could accelerate the discovery of genomics-driven bioactive natural products [76]. Using the established strategy, the enhanced production of the compound microperfuranone was achieved by targeting the native nonribosomal peptide synthetase-like (NRPS-like) gene *micA* in *A. nidulans*.

### 3.2. Aspergillus Niger

*A. niger* is a well-established industrial cell factory that can produce organic acids and a variety of industrial enzymes. Its extraordinary tolerance to extremely acidic environments and ability to hydrolyze a wide range of polymeric substances make it a suitable cell factory for diverse biotechnological applications. The development of CRISPR/Cas9-based genome editing techniques, including multi-gene editing, traceless gene editing, and fine regulation of gene expression, provides a powerful tool for studying gene function and constructing and optimizing cell factories in *A. niger*. Recently, the CRISPR/Cas9 method combined with synthesized sgRNA in vitro was used to disrupt genes involved in galactaric acid catabolism, allowing for efficient galactaric acid production in *A. niger* [53]. This was the first time that CRISPR/Cas9 technology was successfully used for metabolic engineering in *A. niger*. Subsequently, using the same CRISPR/Cas9 strategy, the effective deletion of *gluD*, which encodes an NADPH requiring 2-keto-L-gulonate reductase involved in D-glucuronic acid catabolism, resulted in the accumulation of 2-keto-L-gulonate in the liquid cultivation [77]. Likewise, Kuivanen et al. [78] also demonstrated that the disruption of the *gluF* gene by CRISPR/Cas9 in *A. niger* caused the strain to lose its ability to catabolize D-glucuronate. These findings suggest that the CRISPR/Cas9-mediated genome editing approach has been successfully used to investigate unexplored metabolic pathways and functional genes in *A. niger*. On this basis, an optimized CRISPR/Cas9 method based on Cas9/gRNA RNP complexes assembled in vitro was further developed, which achieved 100% targeting efficiency for single genome editing [64,79]. This approach has also been proven to be suitable for metabolic engineering application of multiplexed genome editing with two or three genomic targets, resulting in increased galactarate production in *A. niger* [64]. In *A. niger*, a Cas9 mutant (D10A nickase), fused with a rat cytidine deaminase, has been exploited for single-base editing, which might result in high-frequency CT substitution at the target sites. This Cas9 mutant is an inactivated nuclease that does not generate DNA DSBs, thus preventing unnecessary deletion or insertion. This newly developed base editing system provides a more convenient tool for studying gene function through targeted genetic alteration [57]. *A. niger*, as a cell factory, is used to produce a variety of proteins and organic acids, and protein secretion is commonly linked to mycelial growth. CRISPR-based genome editing was used to examine the association between protein secretion and filamentous growth by placing the inducible Tet-on conditional expression system upstream of related genes such as *ageB*, *secG*, and *geaB* in studies [80]. The Tet-on system, which employs sophisticated conditional gene expression, can reawaken the biosynthesis of natural products in *A. niger*. The CRISPR/cas9 genome editing strategy, in combination with the Tet-on system, may provide a new approach to enhance protein and organic acid production. As mentioned above, using the CRISPR/Cas9-based genome editing techniques, more experiments on the production and research of enzyme preparations (e.g., pectinases, trehalase, etc.) [75,81,82] and natural metabolites (e.g., citric acid, succinic acid, etc.) [11,83] were conducted in *A. niger.* For example, *A. niger* naturally secretes pectinases to degrade pectin, one of the main carbon sources for filamentous fungi, and W361R mutation in the transcriptional activator GaaR caused by CRISPR/Cas9 leads to constitutive production of pectinases [81]. In another study, *Myceliophthora thermophila* thermostable trehalase (MthT), which can catalyze the hydrolysis of the non-reducing disaccharide trehalose, was heterologously high-expressed in *A. niger* using a CRISPR/Cas9-mediated multi-copy knock-in expression strategy, with the yield reaching 1698.83 U/mL. The addition of the recombinant MthT into 30% starch saccharification liquid greatly boosted the ethanol conversion rate in ethanol fermentation [75]. In experiments with natural metabolite production, the genome editing method disrupted *pyrG,* which encodes the orotidine-5-decarboxylase, resulting in a 2.17-fold increase in citric acid production compared to the control, suggesting that inhibition of uridine/pyrimidine synthesis could promote citric acid overproduction [83]. In addition, the well-established RNP-based CRISPR/Cas9 system has been successfully used in *A. niger* genetic engineering, and significantly improved the succinic acid production by disrupting and overexpressing multiple relevant genes in the engineered strain [11]. Recently, with the improvement of CRISPR/Cas9-based genome editing strategies, an increasing number of studies on gene function and metabolic regulation have been completed in *A. niger* [84,85].

### 3.3. Aspergillus Oryzae

*A. oryzae*, as an important strain in the traditional fermentation and food processing industries, has been well studied and utilized. *A. oryzae* has been known to have a strong ability to secrete large amounts of hydrolytic enzymes, and this property has been widely exploited in the production of recombinant proteins and secondary metabolites [86]. In recent years, the CRISPR/Cas9 system, a versatile genomic editing technology, has been rapidly developed in *A. oryzae* to better adapt to its application in industrial production [87]. Katayama et al. [48] were the first to establish CRISPR/Cas9-based genome editing in *A. oryzae* successfully. In this study, they constructed the plasmids expressing the codon-optimized *cas9*, in which an SV40 nuclear localization sequence was fused to both the N- and C-terminus of the *cas9* gene. The resulting transformed strains have a mutation rate of 10–20%, with most of the mutations being 1-bp deletion or insertion. On this basis, by examining the deletion effect of an *ecdR* gene linked with sclerotial formation [88], it was demonstrated that mutation of *ligD*, a DNA ligase gene involved in NHEJ, significantly enhanced the targeting efficiency of the CRISPR/Cas9 system in *A. oryzae* industrial strains [68]. In addition, an improved *A. oryzae* CRISPR/Cas9 approach, which allows for effective multiple gene deletion or introduction, was well established by recycling AMA1-based genome editing plasmids bearing the drug resistance marker *ptrA* [58]. When a circular donor DNA is provided, this approach greatly boosts HDR-mediated genome editing efficiency. In addition, an instantaneous genome editing technique based on cas9-gRNA RNP complex assembled in vitro has also been successful established in *A. oryzae* [89].

Using these developed genome editing techniques, a variety of basic and production application research was further attempted in *A. oryzae* industrial strains. For instance, adalimumab, a human anti-TNFα antibody, was produced by fusing it with AmyB, a α-amylase. Then, CRISPR/Cas9-based genome editing was used to delete the *Aooch1* encoding a key enzyme of hyper-mannosylation process, to assess the recombinant antibody’s capacity to bind to FcγRIIIa [90]. This genome editing system was used to investigate the functional characterization of glycerol dehydrogenase, revealing that AoGld3, a glycerol dehydrogenase, is involved in the production of the secondary metabolite kojic acid by influencing the expression of *kojA* (an enzyme gene) and *kojR* (a transcription factor gene) involved in the kojic acid biosynthesis [91]. Moreover, using the CRISPR/Cas9 technology, single and double gene disruption of two intracellular triacylglycerol lipases, AoTgla and AoTglb, revealed that disfunction of either AoTgla or AoTglb improved total lipid contents, particularly in the triacylglycerol (TAG) fraction [92]. The biosynthesis of oligopeptides with functional activities has become a research hotspot. In a recent study, promoter exchange of the ACV synthetase (a non-ribosomal peptide synthase (NRPS)) gene (*acv*), was implemented by CRISPR/Cas9-based genome editing for bioactive oligopeptide production in *A. oryzae* [93].

### 3.4. Aspergillus Fumigatus and Other Aspergillus Species

This CRISPR/Cas9 approach has been successfully applied not only to metabolic engineering of the above industrial fermentation strains, but also to the gene manipulation of human pathogenic fungus *A. fumigatus* and other *Aspergillus* species. In *A. fumigatus*, the *pksP* gene, which is required for melanin production, was used as a case study to initially validate the genome editing efficiency of the CRISPR/Cas9 system [94]. On this foundation, a high-efficiency CRISPR genome editing method was established, which carries out precise in-frame integration with an accuracy of 95–100% using an extremely short (about 35-bp) homologous arm (microhomology-mediated end joining, MMEJ) [26]. Using the MMEJ-mediated approach, an exogenous *GFP*, *pksP* (a conidial melanin gene), and *cnaA* (a catalytic subunit of calcineurin gene) were precisely integrated and edited at multiple expected sites. Trypacidin is one of the natural components of the opportunistic human pathogens produced by *A. fumigatus.* Cas9-mediated gene editing was successfully exploited for the functional reconstitution of *tynC*, encoding a polyketide synthase of the trypacidin biosynthetic pathway in a nonproducing *A. fumigatus* strain [95]. Triazole antifungal drugs are indispensable in the clinical treatment of invasive aspergillosis, and triazole-resistant *A. fumigatus* is recognized as a global health issue. Generally, triazole resistance generated by Cyp51A specific amino acid substitution exhibits a typical pattern depending on the mutation site. In a recent study, Cyp51A and Hmg1 mutations that contribute to atypical triazole resistance were assessed using the established RNP-based CRISPR/Cas9 approach in *A. fumigatus* [96,97]. Then, in another study of antifungal drugs, researchers used the RNP-based CRISPR/Cas9 system to disrupt genes encoding putative protein kinases in *A. fumigatus* to identify the genes required for fungal survival under the stress of echinocandin, an antifungal with a limited effect on invasive aspergillosis [98]. Surprisingly, the identified protein kinases were found to be necessary for both hyphal septation and *A. fumigatus*’s capacity to invade lung tissue. In addition to these industrially important strains and pathogenic strains, this versatile genetic manipulation tool has also been successfully established and applied to other *Aspergillus* species, including *A. carbonarius*, *A. novofumigatus*, and *A. terreus*, among others [99,100,101,102]. More studies on the development and utilization of the CRISPR/Cas9 genome editing technology in *Aspergillus* are summarized in Table 1.

**Table 1:** The development and application of the CRISPR/Cas9-based genome editing system in *Aspergillus* species.

Species	Cas9 Expression	gRNA Expression	Delivery Method	DNA Repair System	Gene Editing Type	Efficiency	References
	(Selection Marker, Promoter)	(Promoter)					
*Aspergilli*	*pyrG/argB/hph/ble*, *tef1*	*gpdA*	PMT	NHEJ	1–84 bp deletion or insertion	Success	[63]
*Aspergilli*	*argB/pyrG*, *gpdA*	*U6*, *U3*	PMT	HDR	Multiple-gene disruption	10–100%	[50]
*Aspergilli*	*pyrG*, *tef1*	*U3*	PMT	HDR	gene disruption	80%	[62]
*A. nidulans*	*pyrG*, *gpdA*	*U3*	PMT	HDR	gene activation (gene replacement)	Success	[76]
*A. niger*	*pyrG/hph*, *tef1*	in vitro transcription	PMT	HDR	gene disruption	37.5–100%	[53]
*A. niger*	*hph, tef1*	in vitro transcription	PMT	HDR	gene disruption	Success	[77]
*A. niger*	*hph*, *tef1*	*gpdA*	PMT	HDR	insertion-deletion mutation	100%	[52]
*A. niger*	*hph*, *tef1*	*gpdA*	PMT	NHEJ	short insertions or deletions	Success	[78]
*A. niger*	*amdS*, *glaA*	*U6*	PMT	NHEL/HDR	gene disruption	79%	[51]
*A. niger*	*pyrG*, *pkiA*	tRNA promoter	PMT	NHEJ/HDR	gene disruption/gene replacement	13–97%	[55]
*A. niger*	*hph*, *tef1*	in vitro transcription	PMT	HDR	gene knock-in	Success	[80]
*A. niger*	*hph*, *tef1*	in vitro transcription	PMT	HDR	gene knock-in	Success	[103]
*A. niger*	*hph*, *tef1*	tRNA promoter	PMT	HDR	single/multiple gene knock-out	38–100%	[104]
*A. niger*	*hph*, *tef1*	in vitro transcription	PMT	HDR	gene knock-out	100%	[105]
*A. niger*	*hph*, *tef1*	in vitro synthesis	PMT	HDR	gene knock-in (base editing)	Success	[81]
*A. niger*	RNP		PMT	HDR	single/multiple gene knock-out	100%	[64]
*A. niger*	(rAPOBEC1-nCas9D10A) *hph, tef1*	*U6*	PMT	NHEJ	single base editing	47.4–100%	[57]
*A. niger*	*amdS*, *glaA*	5S rRNA	PMT	NHEJ/HDR	gene disruption	100%	[54]
*A. niger*	*pyrG*, *glaA*	*U6*	PMT	HDR	gene knock-out/knock-in	13.5–54.5%	[59]
*A. niger*	*hph*, *glaA*	5S rRNA	PMT	HDR	gene disruption	100%	[83]
*A. niger*	*hph*, *tef1*	*U6*	PMT	HDR	gene knock-out/knock-in	Success	[75]
*A. niger*	RNP		PMT	NHEJ/HDR	gene knock-out/knock-in	8.3–37.5%	[11]
*A. niger*	RNP		PMT	NHEJ	gene disruption	Success	[106]
*A. niger*	*pyrG*, *pkiA*	tRNA^Pro1^	PMT	HDR	base editing	Success	[107]
*A. niger*	*hph*, *tef1*	*U6*	PMT	HDR	gene knock-in	Success	[82]
*A. niger*	*hph*, *tef1*	glutamine (*gln*) tRNA	Shock wave /PMT	NHEJ/HDR	gene disruption/gene knock-in	Success	[74]
*A. niger*	RNP		PMT	HDR	gene disruption	100%	[79]
*A. niger*	*pyrG*, *pkiA*	tRNA^Pro1^	PMT	HDR	gene knock-out	Success	[108]
*A. niger*	RNP		PMT	HDR	gene replacement	>90%	[109]
*A. niger*	RNP		PMT	NHEJ/HDR	gene knock-out	Success	[84]
*A. niger*	*hph*, *tef1*	in vitro transcription	PMT	HDR	gene knock-in	Success	[110]
*A. niger*	*hph*, *tef1*	tRNA promoter	PMT	HDR	gene knock-out	Success	[85]
*A. oryzae*	*niaD*, *amyB*	*U6*	PMT	NHEJ	1–22 bp deletion or insertion	10–20%	[48]
*A. oryzae*	*niaD*, *amyB*	*U6*	PMT	NHEJ	1–23 bp deletion	100%	[68]
*A. oryzae*	*ptrA*, *amyB/tef1*	*U6*	PMT	HDR	Single/double-gene disruption	50–100%	[58]
*A. oryzae*	*niaD*, *amyB*	*U6*	PMT	HDR	gene disruption	Success	[90]
*A. oryzae*	*pyrG*, *TEF1*	*U6*	PMT	HDR	gene disruption	Success	[92]
*A. oryzae*	RNP		PMT	HDR	gene disruption	56–100%	[89]
*A. oryzae*	*pyrG*, *TEF1*	*U6*	PMT	HDR	Promoter exchange	Success	[93]
*A. fumigatus*	*hph*, *tef1*	*snr52*	PMT	NHEJ/HDR	gene disruption	25–53%	[94]
*A. fumigatus*	*pyr4*, *niiA/gpdA*	*U6-1/2/3* promoters or in vitro transcription	PMT	HDR	Single/double-gene disruption	95–100%	[26]
*A. fumigatus*	*pyrG/hph*, *tet^ON^*	*gpdA*	PMT	NHEJ/HDR	gene disruption/gene replacement	Success	[95]
*A. fumigatus*	RNP		PMT	HDR	gene disruption	97%	[65]
*A. fumigatus*	*hph*, *tef1*	*gpdA*	PMT	HDR	base editing	Success	[111]
*A. fumigatus*	RNP		PMT	HDR	gene knock-in	Success	[97]
*A. fumigatus*	RNP		PMT	HDR	gene disruption/gene replacement	93%; 10–20%	[112]
*A. fumigatus*	RNP		PMT	HDR	gene replacement	Success	[96]
*A. fumigatus*	RNP		PMT	HDR	gene disruption	90%	[98]
*A. carbonarius*	*hph*, *tef1*		AMT	NHEJ/HDR	Single-gene disruption	27%	[99]
*A. carbonarius*	RNP		PMT	HDR	gene disruption	Success	[100]
*A. novofumigatus*	*pyrG*, *tef1*	*gpdA*	PMT	HDR	gene disruption	Success	[101]
*A. terreus*	*pyrG*, *gpdA*	5S rRNA promoter	PMT	HDR	gene disruption	71%	[113]
*A. lentulus*	RNP		PMT	HDR	gene knock-in	Success	[102]

RNP, in vitro-assembled Cas9 and gRNA ribonucleoprotein complexes; PMT, a polyethylene glycol (PEG)/CaCl2-mediated protoplast transformation system; AMT, *Agrobacterium tumefaciens*-mediated transformation system.

## 4. Conclusions

The CRISPR/Cas9 system is a powerful genome editing tool that has been used on a variety of industrially important and pathogenic *Aspergillus* species, including *A. nidulans*, *A. oryzae*, *A. niger*, and *A. fumigatus*. However, in order to create effective CRISPR/Cas9-mediated genome editing strategies for *Aspergillus* species, various restrictions and hurdles must be overcome. Off-target effects generated by Cas9’s non-targeted nuclease activity are a key barrier in genome editing of *Aspergillus* employing CRISPR technology.

As a result, a variety of strategies are employed to reduce the likelihood of off-target effects. The sgRNA sequence, which is crucial for Cas9 nuclease activity, should be carefully designed to avoid nucleotide mismatches with non-targeted sites in the genome. After successful genome editing, the *cas9* gene should be regulated by selecting appropriate promoters to prevent its further expression, or transient expression should be achieved through Cas9/sgRNA RNP complexes assembled in vitro. As a result, off-target effects might be reduced in *Aspergillus* species by limiting Cas9 expression and activity, designing stable and unique gRNAs. In addition, the efficiency of multi-gene editing in *Aspergillus* is determined by the design of multiple sgRNA expression cassettes and the efficacy of co-transformation. The development of genome editing technology based on the CRISPR/Cas9 system will dramatically simplify genetic manipulation, and substantially improve the research of functional genes as well as the production of recombinant proteins and natural products in *Aspergillus* species.

## Data Availability

Not applicable.
